# Integration of multiomics data shows down regulation of mismatch repair and tubulin pathways in triple-negative chemotherapy-resistant breast tumors

**DOI:** 10.1186/s13058-023-01656-x

**Published:** 2023-05-24

**Authors:** Xiaojia Tang, Kevin J. Thompson, Krishna R. Kalari, Jason P. Sinnwell, Vera J. Suman, Peter T. Vedell, Sarah A. McLaughlin, Donald W. Northfelt, Alvaro Moreno Aspitia, Richard J. Gray, Jodi M. Carter, Richard Weinshilboum, Liewei Wang, Judy C. Boughey, Matthew P. Goetz

**Affiliations:** 1 Department of Quantitative Health Sciences, Mayo Clinic, Rochester, MN USA; 2 Department of Surgery, Mayo Clinic, Jacksonville, FL USA; 3 Department of Medical Oncology, Mayo Clinic, Scottsdale, AZ USA; 4 Department of Medical Oncology, Mayo Clinic, Jacksonville, FL USA; 5 Department of Surgery, Mayo Clinic, Scottsdale, AZ USA; 6 Department of Pathology, Mayo Clinic, Rochester, MN USA; 7 Department of Molecular Pharmacology and Experimental Therapeutics, Mayo Clinic, Rochester, MN USA; 8 Department of Surgery, Mayo Clinic, Rochester, MN USA; 9 Department of Medical Oncology, Mayo Clinic, Rochester, MN USA

## Abstract

**Background:**

Triple-negative breast cancer (TNBC) is the most aggressive breast cancer subtype. Patients with TNBC are primarily treated with neoadjuvant chemotherapy (NAC). The response to NAC is prognostic, with reductions in overall survival and disease-free survival rates in those patients who do not achieve a pathological complete response (pCR). Based on this premise, we hypothesized that paired analysis of primary and residual TNBC tumors following NAC could identify unique biomarkers associated with post-NAC recurrence.

**Methods and results:**

We investigated 24 samples from 12 non-LAR TNBC patients with paired pre- and post-NAC data, including four patients with recurrence shortly after surgery (< 24 months) and eight who remained recurrence-free (> 48 months). These tumors were collected from a prospective NAC breast cancer study (BEAUTY) conducted at the Mayo Clinic. Differential expression analysis of pre-NAC biopsies showed minimal gene expression differences between early recurrent and nonrecurrent TNBC tumors; however, post-NAC samples demonstrated significant alterations in expression patterns in response to intervention. Topological-level differences associated with early recurrence were implicated in 251 gene sets, and an independent assessment of microarray gene expression data from the 9 paired non-LAR samples available in the NAC I-SPY1 trial confirmed 56 gene sets. Within these 56 gene sets, 113 genes were observed to be differentially expressed in the I-SPY1 and BEAUTY post-NAC studies. An independent (*n* = 392) breast cancer dataset with relapse-free survival (RFS) data was used to refine our gene list to a 17-gene signature. A threefold cross-validation analysis of the gene signature with the combined BEAUTY and I-SPY1 data yielded an average AUC of 0.88 for six machine-learning models. Due to the limited number of studies with pre- and post-NAC TNBC tumor data, further validation of the signature is needed.

**Conclusion:**

Analysis of multiomics data from post-NAC TNBC chemoresistant tumors showed down regulation of mismatch repair and tubulin pathways. Additionally, we identified a 17-gene signature in TNBC associated with post-NAC recurrence enriched with down-regulated immune genes.

**Supplementary Information:**

The online version contains supplementary material available at 10.1186/s13058-023-01656-x.

## Background

It is estimated that over 2.2 million breast cancer cases occurred worldwide in 2020, representing 1 in 4 cancers diagnosed among women [1, 2]. The molecular classification of breast cancer is based on human epidermal growth factor receptor-2 (HER2) gene amplification and the expression of sex hormones, estrogen, and progesterone receptors. Triple-negative breast cancer (TNBC), approximately 15% of invasive breast cancers, is the most aggressive breast cancer subtype; it lacks estrogen receptor (ER) expression, progesterone receptor (PR) expression, and HER2 gene amplification [3, 4]. Patients with TNBC have the highest mortality rates compared to the other breast cancer subtypes and are treated with chemotherapy prior to surgery, known as neoadjuvant chemotherapy (NAC) [5, 6]. NAC reduces the tumor burden and nodal involvement. Most importantly, the response of TNBC to NAC is associated with long-term patient outcomes [7]. However, nearly 50% of patients with TNBC have residual disease after NAC [6]. Of those, 20–30% of TNBC patients develop early disease progression within three years and exhibit high metastatic recurrence rates and poor long-term outcomes [7, 8].

With advances in high-throughput technologies, large-scale breast cancer genomics and transcriptomics data have been collected and analyzed to identify biomarkers associated with prognosis as well as treatment resistance [7, 9–16]. Most TNBC studies have focused on subtyping or developing signatures associated with prognosis or disease recurrence, mainly utilizing pretreatment gene expression data from tumor samples. Few studies evaluated transcriptomics data from pre-NAC and post-NAC TNBC tumors [7, 13–16]. These studies have examined changes in gene expression post-NAC to understand the molecular underpinnings driving treatment resistance in TNBCs. Identifying genes involved in chemotherapy-resistant disease (residual disease post-NAC) could lead to better stratification of patients and better therapeutic strategies for TNBC. Balko et al. measured gene expression for 450 transcripts from pre- and post-NAC breast tumor issues using the NanoString platform and identified DUSP4 deficiency as an important mechanism of TNBC drug resistance [15]. The I-SPY1 breast cancer clinical trial study by Magbanua et al. has shown an association of response with cell cycle and immune pathways [13]. Hancock et al. generated pre- and post-transcriptomics data using the Ion Torrent platform. They identified an association of increased SMAD2 expression, TP53 loss, and MYC-driven amplification with chemorefractory TNBC tumors [7]. Importantly, the I-SPY1 study and Hancock et al.'s study are complementary in that they describe the depletion of the immune microenvironment and up regulation of markers related to stemness in chemoresistant tumors [7, 13].

In our study, we investigated the topological differences in chemoresistant TNBC tumors. Specifically, we investigated transcriptome sequencing data from treatment resistant TNBC tumors. Since TNBC represents a heterogeneous group of TNBC subtypes, we focused on androgen receptor (AR) negative TNBC (i.e., ER-, PR-, HER2- and AR-). We developed a signature associated with early recurrence using post-NAC gene expression data from chemoresistant TNBC tumors. That signature was then compared with the patient's pre-NAC transcriptomics, whole-exome sequencing (WES), and reverse-phase protein array (RPPA) data. Moreover, we validated the signature using I-SPY1 pre- and post-NAC TNBC data. Finally, we confirmed that the 17-gene signature could predict post-NAC patients with an elevated risk of recurrence.

## Methods

### Materials or datasets

#### A chemorefractory cohort from the Mayo Clinic breast cancer study

The Breast Cancer Genome Guided Therapy Study (BEAUTY) is a prospective institutional review board–approved NAC clinical trial (NCT02022202) from March 2012 to May 2014. As previously described [6], the BEAUTY study enrolled 140 patients with invasive breast cancer across Mayo Clinic Rochester, Mayo Clinic Florida, and Mayo Clinic Arizona. All patients provided written informed consent. The analysis in this manuscript includes the subcohort of 42 patients with TNBC who received 12 weeks of weekly paclitaxel, followed by four cycles of an anthracycline-based regimen. The methods were performed in accordance with relevant guidelines and regulations and approved by the Mayo Clinic Institutional Review Board (IRB). Pathological complete response (pCR) was defined as the absence of invasive tumors in the breast and axillary lymph nodes. Residual disease (RD) was defined as either pCR = No, or RCB at surgery = 1 or higher, and/or overall cancer cellularity (pathologist) > 20.

Eighteen of the 42 patients with TNBC had surgical collections taken after NAC intervention. We obtained pre-NAC and post-NAC transcriptomics data for these 18 cases (36 tumors) and identified luminal androgen positive (LAR) cases using our in-house algorithm (LAR-Sig) [17]. Due to significant clinical, molecular, and pathological differences we previously observed between LAR and non-LAR tumors, we focused our analysis on 15 non-LAR TNBC patients [18–22]. We also removed three samples as the post-NAC biopsies appeared to collect tumor bed tissues with the samples presenting with only minimal epithelial tissue (see below). The final cohort for paired visit analysis included 12 paired samples (Additional file 2: Table s1) with a median post-NAC follow-up of 4.7 years, including four early recurrent (ERC, < 24 months post-NAC) and eight nonrecurrent samples (NRC, > 48 months post-NAC, follow-up time of 4.5 to 6.0 years). None of the 12 patients in this investigation were lost to follow-up.

#### BEAUTY TNBC data cohort

Genomic and transcriptomic data from the BEAUTY TNBC cohort have been described in details in Goetz et al. [6] and are available through the dbGap ID phs001050.v1.p1. Briefly, the WES sequencing data were aligned and processed against the human genome (hg19) through Mayo Clinic’s DNA sequencing analysis pipeline, GenomeGPS 1.0.1, following Broad GATK variant discovery best practices (version 3) of alignment, realignment and recalibration, and multi-sample joint genotyping [23]. Somatic copy number alterations were called by PatternCNV version 1.0.1 [24]. The base-2 logarithm ratio (LogR) of the coverage between a tumor sample and the reference set was computed for each 100 bp window. A threshold of |LogR|> 0.5 was used to call a region amplified or deleted. Paired-end RNA-seq data alignment and gene expression quantification was performed using the MAP-RSeq pipeline version 2.0.1 against hg19. Normalization of the gene expression was performed using Conditional Quantile Normalization [25]. Here we summarize the findings of our investigation of paired gene expression data, paired protein expression data (described below), and pre-NAC WES data from the BEAUTY cohort.

#### Reverse-phase protein array data from the BEAUTY breast cancer study

Lysates from paired tissue preparations of the paired breast tumor tissue samples at pre-NAC and post-NAC time points from the BEAUTY study were provided for evaluation by reverse-phase protein array (RPPA). The relative protein levels were determined by interpolation using a super curve provided by MD-Anderson Cancer Center for functional proteomics [26]. Among the twelve patients in the ERC and NRC groups, four had only pre-NAC RPPA data, two had only post-NAC RPPA data, four had both pre- and post-NAC data, and two did not have any pre or post RPPA data (please see Additional file 2: Table s1 for further details). We performed two-sample Student's t-tests with pooled variances on the log2-transformed protein levels for 295 antibodies between the pre-NAC and post-NAC data.

#### Tumor bed assessment using digital deconvolution

A digital deconvolution was performed on the post-NAC samples using gene expression data to ensure sufficient epithelial cells were obtained rather than tumor beds. In a single-cell RNA sequencing experiment of 24,271 cells, representing twenty cell types from five patients (average of 4854 cells per patient) with primary TNBC were previously identified. Data was integrated and canonical correlation analysis among the tumor samples was performed with Seurat [27]. We obtained the single-cell gene expression counts and t-SNE clustering scheme and constructed a balanced dataset with prevalent and low-abundance cell types. Our balanced dataset consisted of eighty nearest neighbor cells to each of the twenty centroids to adequately represent the least prevalent cell type (i.e., 106 immature perivascular-like fibroblasts, imPVL [28]. The deconvolution was implemented with the twenty cell types merged into nine lineages. Each BEAUTY TNBC post-NAC study sample with bulk RNA-Seq and phenotype data (*n* = 18) was individually evaluated with pseudo-replication to achieve a sampling set larger than the nine cell types. We preferred a reasonably strong correlation (Spearman’s) between the cellular estimates from our deconvolution model and the pathological assessment of in situ cancer cellularity of the post-NAC surgical specimens, as well as tumor microenvironment contribution scores (immune and stromal) from the ESTIMATE package [29]. As a result, we identified three RNA-Seq samples (two nonrecurrent and one early recurrent) presenting predominately as biopsies of tumor bed (i.e., 15 were left for further analysis), removing them along with their pre-NAC counterpart.

#### I-SPY1 trial gene expression data

The clinical and molecular data from the I-SPY1 breast cancer trial have been described in detail previously [30, 31]. The normalized expression data were obtained from the Gene Expression Omnibus (GEO) database using the accession ID: GSE32603 [13]. From the I-SPY1 GEO dataset (*n* = 141, with baseline data), we selected patients with TNBC (*n* = 39, 27.7%) that were resistant to NAC (*n* = 23, 59.0%). Similar to the BEAUTY breast cancer study, we only considered the non-LAR TNBC samples in the I-SPY cohort (*n* = 9, 39.1%) having paired gene expression data at both pre-NAC treatment (T1) and post-NAC or surgery (TS) time points. Furthermore, the samples were confirmed to be non-LAR using our recently published method (the LAR-Sig [17]); additional PAM50 analysis confirmed that all samples were also basal-like. Among the I-SPY1 cohort there were *n* = 5 patients who had early recurrence (less than two years after NAC) and *n* = 4 patients who remained nonrecurrent for more than four years for gene expression data analysis (Additional file 2: Table s2).

#### Sample specific gene set variation analysis using gene expression data

Sample-wise pathway analysis of the RNA-Seq data was carried out using the gene set variation analysis (GSVA) method [32] for the curated (C2, *n* = 2232) and hallmark (H, *n* = 50) gene sets from MSigDB version 7.1 [33]. GSVA scores were calculated for the pre-NAC and post-NAC gene expression datasets. The scores were then evaluated using linear regression with empirical Bayes statistics with a limma package [34]. Gene sets were determined to be significant with a *p*-value < 0.05. Gene sets and individual genes were filtered based on the scores, fold-changes, and *p*-values observed in the I-SPY1 and BEAUTY trials.

#### Statistics and bioinformatics analysis

For gene expression data generated with RNA-seq (e.g., the BEAUTY cohort and TCGA), differential expression (DE) analysis was performed using the empirical Bayes quasi-likelihood F-test (QLF) to identify genes associated with recurrence for both the pre-NAC and post-NAC data with edgeR [35]. For other gene expression data sets generated with microarray (e.g., the ISPY1 cohort), DE analysis was performed with R package limma using linear modeling with empirical Bayes methods [34]. Over-representation analysis (ORA) was performed to identify cytobands significantly enriched in the DE genes using WebGestalt [36]. Copy number alterations frequencies associated with recurrence were evaluated with Fisher’s' exact test. Circos plots were generated with Rcircos [37]. Survival analysis for the selected biomarkers was independently performed using the online tool Kaplan–Meier (KM) plotter with the TNBC cohort (n392) and the default threshold of 60-month follow-up [38]. In addition, (1) The Cancer Genome Atlas (TCGA) paired tumor and normal-adjacent TNBC samples [39], (2) non-LAR vs. LAR TNBC gene expression cohorts and (3) non-LAR gene expression cohorts from Thompson et al. [17] with respect to pathologic complete response phenotype were also surveyed using publicly available gene expression data [6, 40–44]*.*

#### Confirmation Cohorts

We investigated several datasets to confirm that the dysregulation of our gene signature originated from non-LAR TNBC. Biomarker Expression profiles were attributed to non-LAR TNBC samples using the paired TCGA TNBC. TCGA breast cancer fastq files were obtained from the TCGA data portal (version November 11, 2013).

Publicly available microarray datasets (GSE106977, GSE25065, GSE25505, GSE32646) were independently normalized and scaled with the ComBat algorithm as described previously [17]. DE analyses of RD versus pCR and non-LAR versus the LAR were performed with R package limma [32].

#### Assessment of biomarkers using machine learning methods

The I-SPY1 and BEAUTY studies were pooled together and scaled with the ComBat algorithm from the sva package (v 3.14.0) [45] to remove the batch effect. Given the lack of sampling power, we implemented the SuperLearner R package [46] to assess the classification ability of the markers using a threefold cross-validation strategy on the intermixed cohort of I-SPY1 and BEAUTY samples. We examined six classification algorithms, including a generalized linear model (glmnet), k nearest neighbors (k-NN), neural networks, random forests, support vector machine (SVM), and kernel SVM algorithms. The models were implemented using down selection methods (Pearson’s correlation, Spearman’s rank correlation, and random forest). Similarly, we evaluated the BEAUTY cohort’s clinical features (age, menopause, TNM stages, and pre-NAC and post-NAC Ki67 scores) using twofold cross-validation with SuperLearner, combined with the biomarkers. We monitored the area under the receiver operating curve (AUC) to indicate the model’s classification performance. Furthermore, we evaluated the Cox proportional hazards of the univariates in both the I-SPY1 and BEAUTY cohorts. In addition to the classification performance of random forests, kNN, and neural networks, we evaluated non-negative least squares (nnls), Bayesian general linear model (glm), and rpart algorithms.

## Results

### Chemoresistant sample selection from the BEAUTY TNBC cohort

TNBC patients enrolled in the prospective BEAUTY clinical trial neoadjuvant study were treated with NAC and were followed up for at least 4 years. We have previously compared pCR and resistant disease in pre-NAC TNBC tumors [6]. We identified several pathways significantly associated with pCR in the TNBC cohort, including the glucocorticoid receptor (GR) regulatory network; *FOXA1*, *FOXA2*, and *FOXA3* networks; integrins; *SMAD2/3*; and androgen receptor signaling. In this study, we focused on the molecular analysis of patients who did not respond to NAC treatment (non-pCR patients—who failed to respond to paclitaxel and anthracycline/cyclophosphamide combination therapy). We aimed to identify genomic signatures associated with recurrence using the pre- and post-NAC treatment data. From the BEAUTY breast cancer NAC study, we analyzed RNA-Seq data from 18 paired (pre-NAC and post-NAC) triple-negative breast tumors. Our previous investigation and existing studies have demonstrated that the luminal androgen receptor (LAR) TNBC tumors are biologically and clinically distinct from non-LAR tumors. Hence, we removed the three TNBC LAR tumor pairs identified by LAR-Sig from further analysis. In addition, tumor bed analysis was performed using an *in-silico* deconvolution method, indicating that three post-NAC biopsies contained low luminal and basal epithelial compositions. Spearman’s correlation to the pathological assessment of in situ cancer cellularity was observed to be 0.53 among basal epithelial cells and 0.50 when considered together (summed) with mature luminal epithelial cells. Similarly, the correlation between our deconvolution model assessment of immune cells and the immune assessment score from the ESTIMATE package was 0.59 and 0.41 for the pre-NAC and post-NAC samples, respectively. While the correlation between our stromal assessment and the ESTIMATE package's stromal assessment score was 0.83 and 0.81 for the pre-NAC and post-NAC, respectively. Hence, we removed those three samples from the analysis. We investigated 12 paired non-LAR TNBC samples with matched pre- and post-NAC gene expression data (Table 1). Of these 12 patients, four had an early recurrence (less than two years after the completion of NAC), and eight remained recurrence-free for more than four years. Furthermore, for these 12 non-LAR TNBC patients, all patients had pre-NAC WES data, and 9/12 had paired protein and phosphoprotein (RPPA) data.

**Table 1:** Demographic and clinical characteristics of TNBC patients included in this analysis

	NRC (*n* = 8)	ERC (*n* = 4)	Total (*n* = 12)
Age			
Mean (SD)	51.4 (11.0)	55.8 (13.1)	52.8 (11.3)
Range	36–70	40–67	36–70
Menopausal status			
Peri	1 (12.5%)	0 (0.0%)	1 (8.3%)
Post	4 (50.0%)	3 (75.0%)	7 (58.3%)
Pre	3 (37.5%)	1 (25.0%)	4 (33.3%)
T stage			
T2	3 (37.5%)	2 (50.0%)	5 (41.7%)
T3	5 (62.5%)	2 (50.0%)	7 (58.3%)
N stage			
N0	6 (75.0%)	3 (75.0%)	9 (75.0%)
N1	2 (25.0%)	1 (25.0%)	3 (25.0%)
M stage			
M0	8 (100.0%)	4 (100.0%)	12 (100.0%)
Race			
NA	0 (0.0%)	1 (25.0%)	1 (8.3%)
American Indian or Alaska Native	1 (12.5%)	0 (0.0%)	1 (8.3%)
Caucasian	7 (87.5%)	3 (75.0%)	10 (83.3%)

**Fig. 1:**
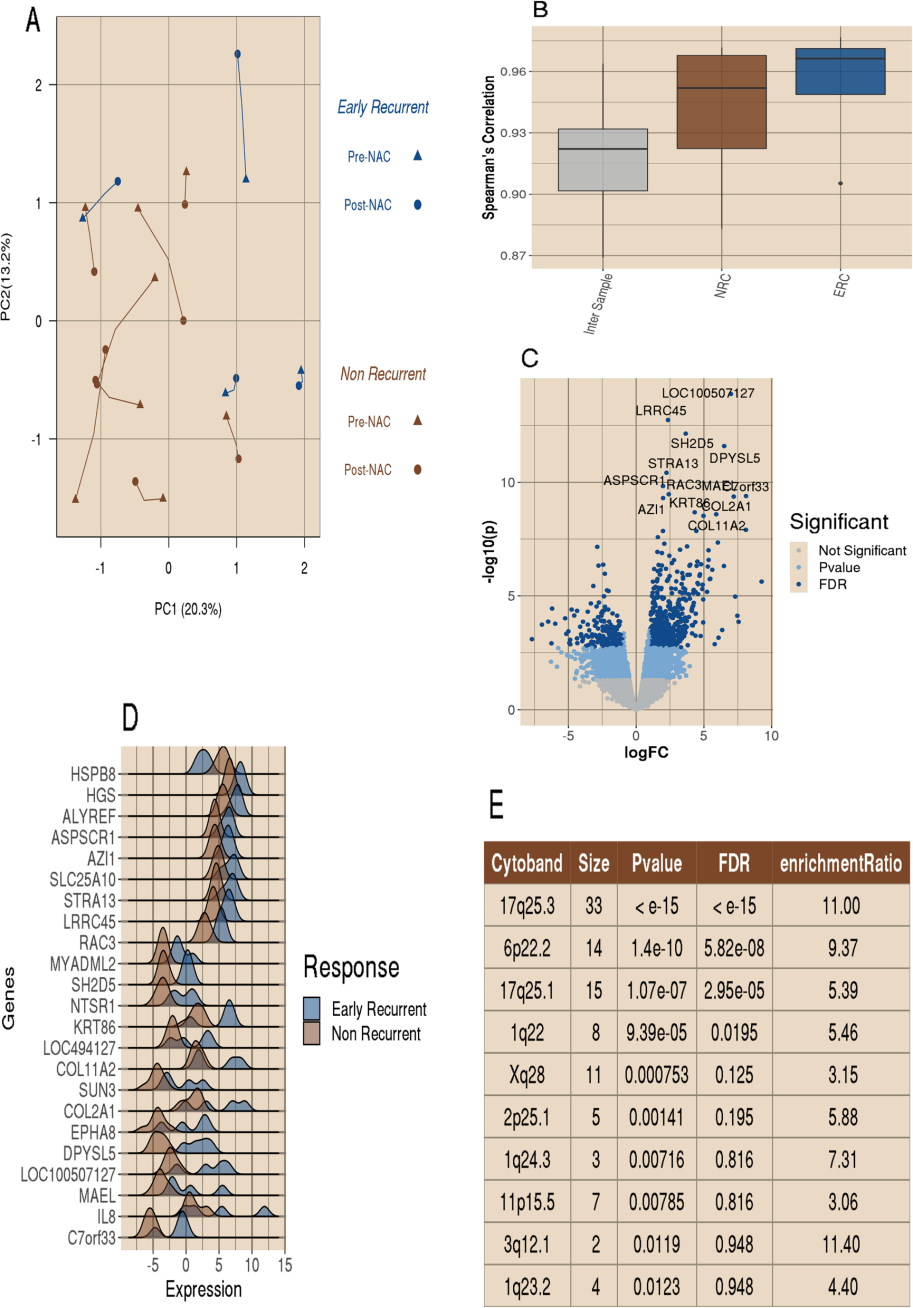
Post-NAC up-regulations were observed in the early recurrence. **A** Co-inertia plot of the top two principal components from the paired bulk RNA-Seq dataset for 12 BEAUTY TNBC non-responders. Lines between points connect paired samples, with pre-NAC visit indicated with a triangular point and post-NAC resection indicated with regular points. The four early recurrent patients are presented in blue, and the eight nonrecurrent patients are presented in brown. The line lengths between sample visits are indicative of the between visit correlation. We observe a slight trend for nonrecurrent samples to shift left on PC1 and early recurrent samples to shift away from the center on PC2. **B** Distribution of the Spearman’s Correlations for inter- and intra- samples. Intra-sample correlations were calculated between pre- and post-NAC expression of the same sample. Inter-sample correlations were calculated for all sample pairs. **C** A volcano plot of the bulk RNA-Seq DE data that compares post-NAC surgical samples of recurrent versus nonrecurrent samples. A total of 20,543 genes were tested, with 2,944 (14.3%,) having a *p*-value < 0.05 and an absolute fold change greater than 2 (indicated with in the light blue), with the majority (1,859, 63.1%) of those being up regulated in the early recurrent samples. There were 660 genes with significant DE after correction for the false discovery rate, indicated with the darker blue. The top 13 most significant genes (with *p*-value < 10^–8^ and FDR < 10^–5^) were labeled, all of which were up regulated in the early recurrent samples. **D** Ridges plot of the twenty-three genes with FDR < 10^–4^. The distribution for the early recurrent tumors is indicated in the dark blue, and the nonrecurrent samples is presented in brown. **E** Results table for the top 10 cytobands enriched in DE genes between early recurrent and nonrecurrent TNBC samples

We evaluated 20,543 genes assayed from both pre-and post-NAC biopsies. A co-inertia plot using the first two principal components of paired samples is presented in Fig. 1A. In the plot, we noted a slight separation trend between patients in the early-recurrence (ERC; *n* = 4) group and the nonrecurrent (NRC; *n* = 8) group for the first principal component. This component explains 20.3% of the variance observed in the pooled dataset. We present the distribution of correlations in Fig. 1B. We observed a higher intra-sample correlation of paired pre- and post-NAC data than the inter-sample correlation within pre- and post- NAC data. Additionally, we see a small notable decrease in intra-sample correlations with the NRC cohort as opposed to the ERC cohort. Our evaluation of the pre-NAC transcriptome gene expression between the ERC and NRC groups identified only 11 genes to be differentially expressed after correcting for multiple testing with a false discovery rate (FDR) < 0.05.

### Post-NAC transcriptomic analysis showed clusters of genes differentially expressed on chromosome arms 1q and 17q

Conversely, the comparison of early-recurrent and nonrecurrent tumors in the post-NAC gene expression data identified 660 genes differentially expressed with at least twofold change and FDR < 0.05 (Fig. 1C, Additional file 2: Table s3). Among these genes, 478 (72.4%) were up regulated in ERC tumors (Fig. 1C). The list of the top 23 significant genes based upon an FDR < 1e-4 is shown in Fig. 1D. Further over-representation analysis (ORA) identified 10 cytobands that were significantly enriched in the 660 DE genes (Fig. 1E), including 17q (17q25.3, 33 genes, *p*-value less than 10^–15^ and 17q25.1, 15 genes, *p*-value 1.01 × 10^–1^), 6p (6p22.2, 14 genes, *p*-value 1.40 × 10^–10^), 10q (10q22.3, 9 genes, *p*-value 6.51 × 10^–5^), and 1q (1q, 8 genes, *p*-value 9.39 × 10^–5^; 1q24.3, 3 genes, *p*-value 0.007; and 1q23.2, 4 genes, *p*-value 0.01).

### Genomic analysis of pre-NAC data confirms amplifications in chromosomes 1q and 17q in the ERC group compared to the NRC group

**Fig. 2:**
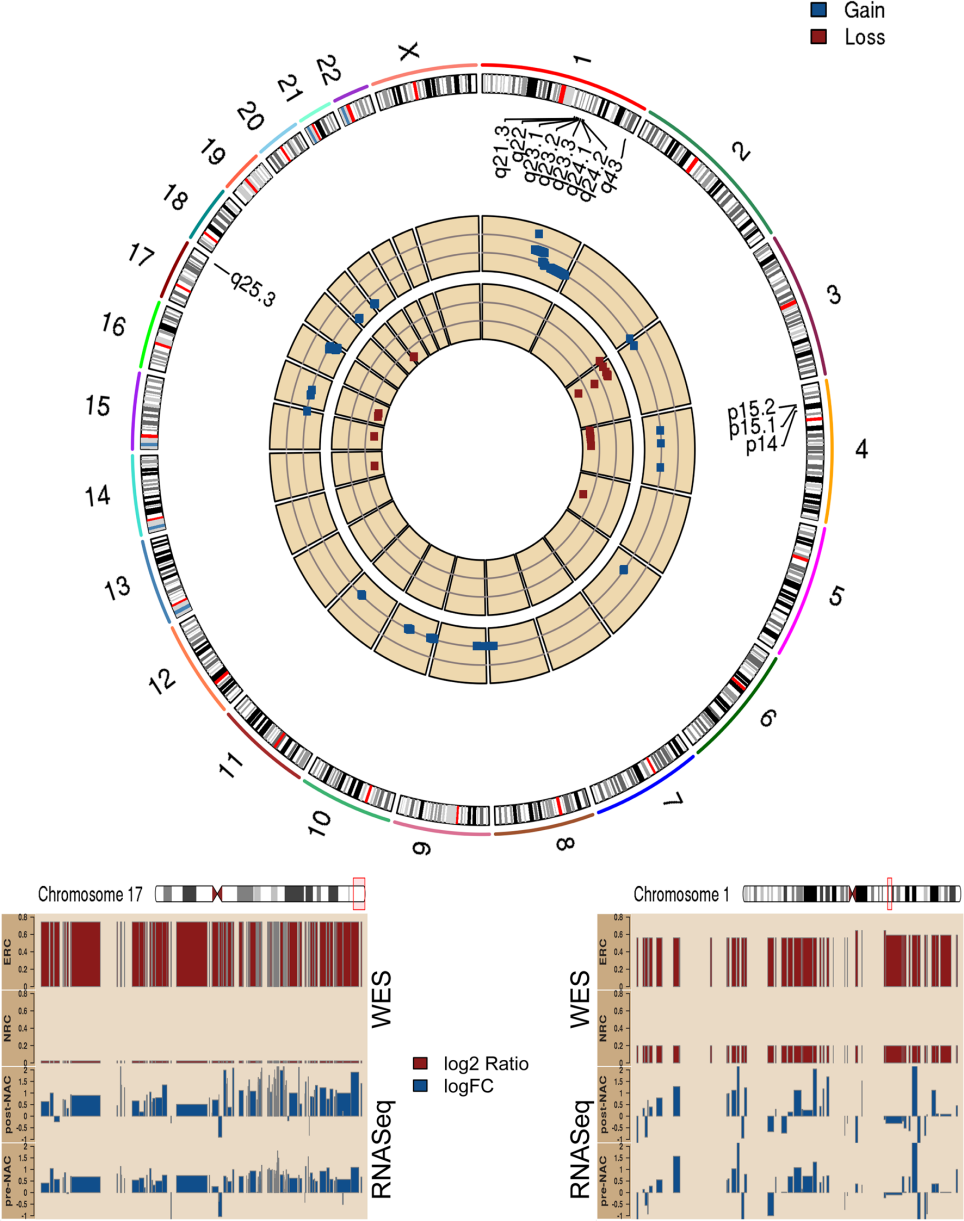
Circos plot of the gene-level copy number alteration. Top: Gene-level CNA events (pre-NAC) that were different between the ERC and NRC group with a *p*-value of less than 0.05 are shown. Cytoband regions that were observed to be over-represented with genomic alterations are labeled, including 17q25, 1q24-24, and 4p15.2-.32. The -log10 p values from the Fisher’s test of the copy gains are presented in the outer track (blue), and the -log10 p values of the Fisher’s test of copy losses are shown in the inner circle (deep red). Gains on chr1q and chr17q were observed in 3 out of 4 recurrent patient tumors. Bottom panels showed two regions (chr17q on the left and chr1q on the right) with copy gains (dark red, top) that were observed only among the ERC group but not in the NRC. Across these two regions, we also observed consistent higher gene expression, presented as logFC for ERC vs NRC (dark blue, bottom)

Since WES data of post-NAC tumors were unavailable, we examined copy number alterations in the pre-NAC patient tumors between the ERC and NRC groups. We identified a common gain/amplification in large regions of the chr1q (q21.3-q24.2,395 altered genes) and chr17q (q25.3, 97 altered genes) in at least 3 out of 4 recurrent patients; however, they were not detected in any of the eight nonrecurrent patients (*p*-value <  = 0.012 by Fisher's exact test). The 1q and 17q gain events involved 492 genes (Additional file 2: Table s4). Similarly, we also identified a deletion of 4 genes (in 4p) among the ERC group. A circos plot depicting the altered copy number regions is presented in Fig. 2. We also present histogram views of the median copy gains in the early-recurrent samples for the 17q and 1q regions (particularly 1q24), along with the elevated expression levels observed with these copy gains using pre-NAC and post-NAC RNA-Seq data (lower panels of Fig. 2, respectively).

### Somatic mutation showed frequent mutations in TP53 and MUC4.

We investigated the somatic mutations in the pre-NAC sample biopsies using WES tumor and blood DNA data. We identified 392 high/moderate impact somatic mutations corresponding to 366 genes in the four early-recurrent patient tumors and 545 high/moderate impact mutations in 519 genes in the eight nonrecurrent patient tumors (Additional file 2: Table s5). Due to the small sample size, low mutation frequency across genes and low overlap among the genes were observed between the ERC and NRC groups. It was noted that the most frequently mutated genes across our cohort were *TP53* (all four ERC samples and five of eight NRC samples) and *MUC4* (two of the four ERC samples and three of eight NRC samples), consistent with a previous publication [47].

### RPPA analysis indicates pre-NAC proteomic differences

We evaluated protein expression (232 proteins) and phosphorylation (63 phosphorylation sites) data obtained from the pre-NAC and post-NAC tumors using RPPA. The pre-NAC RPPA data were compared between the ERC and NRC groups. We observed that 30 proteins were significantly different (*p*-value < 0.05), including ten phosphorylation sites (10/63, 15.9%) and 20 total proteins (20/232, 8.6%) among the pre-NAC samples. In contrast, there were only four protein level variations, and no phosphorylation site differences in the post-NAC RPPA data. See Additional file 2: Tables s6-s7 for the full results.

### Down regulation of Tubulin and up regulation of growth receptors in the nonrecurrent post-NAC tumors

While clinically significant, the investigative niche of our study presented us with a small sampling size challenge. In order to address this challenge, we first sought to validate results from the BEAUTY study with the I-SPY1 clinical trial (the only publicly available study with both pre-NAC and post-NAC gene expression data). However, the biological variation and technical variability of these two studies utilizing two high-throughput platforms (BEAUTY RNA-Seq and I-SPY1 microarray) provided a small number of concordantly differentially expressed genes (see Additional file 1: Fig. 1). In order to overcome the limitations of a small-sample-size study and get greater insight into the groupings of genes (biological pathways/gene sets) from the gene expression data, a parallel single-sample gene set analysis was carried out rather than a single gene expression analysis. Gene set analysis enabled us to minimize the false discovery rate by reducing the n >  > p by a factor of 6, while capturing the global changes rather than specific univariate differences. We conducted Gene Set Variation Analysis (GSVA) and obtained individual gene set scores for all non-LAR TNBC tumors with the pre-NAC and post-NAC RNA-Seq data. We performed three comparisons: 1) post-NAC difference between ERC vs. NRC, 2) paired pre-NAC and post-NAC differences in ERC, and 3) paired pre-NAC and post-NAC differences in NRC for both BEAUTY and I-SPY1. Among the total 2282 gene sets, we identified 251 gene sets (Additional file 2: Table s8a) that were significantly altered by comparing the ERC and NRC groups in the post-NAC tumors (*p*-value < 0.05).

**Fig. 3:**
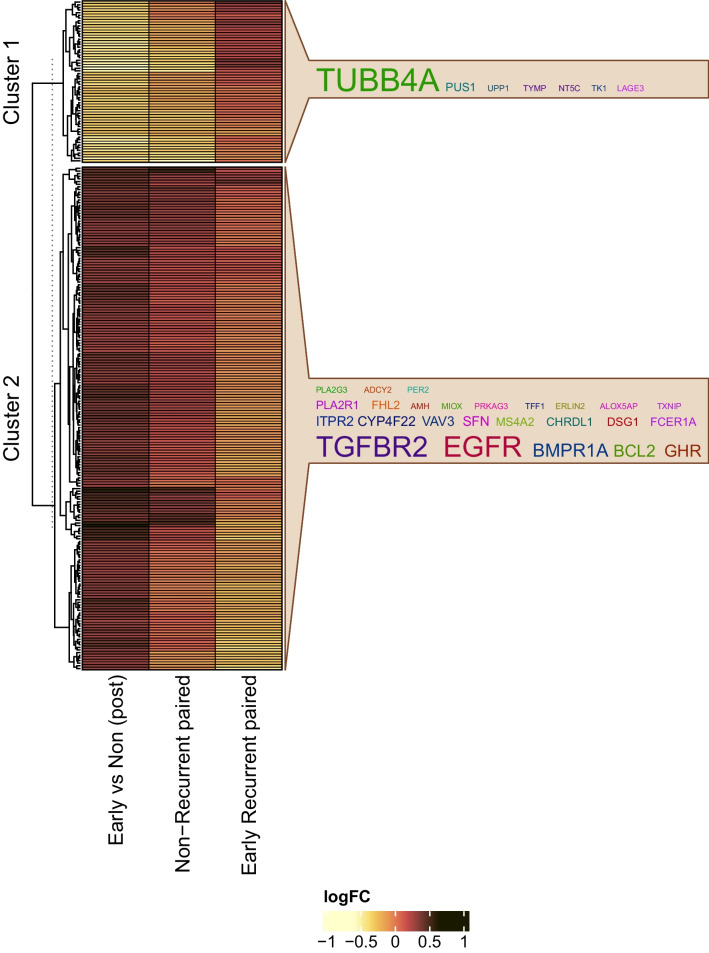
GSVA analysis showed pathway-level/gene set activity differences between the early recurrent and nonrecurrent groups. In the figure, Column 1—Nonrecurrent paired pre-NAC vs. post-NAC), Column 2 – Early recurrent paired pre-NAC vs. post-NAC, and Column 3- post-NAC (early recurrence vs. nonrecurrent). Unsupervised clustering showed two clusters with a distinctive pattern between the two groups (heatmap panel). Cluster 1 gene sets that were upregulated in the early recurrent group included metastasis-promoting gene sets, DNA mismatch repair, and tp53 gene sets. The most commonly observed gene among these gene sets was *TUBB4A*. Cluster 2 included signaling gene sets such as *FOXO*, *TGF*β, and apoptotic known to be associated with tumor suppression. Details of gene set names and cluster assignments can be found in Additional file 2: Table s8a

In addition, we performed a hierarchical clustering analysis of the log2 fold changes for 251 gene sets (Fig. 3). The log fold changes represented the changes in post-NAC data and the differences between the tumors for the ERC and NRC groups. The average silhouette width, a metric of within-class similarity and between-class dissimilarity, was 0.90 for two gene set clusters and was markedly reduced for three gene set clusters (0.61). We observed that the first cluster of 61 gene sets were down regulated in the post-NAC nonrecurrent group of patients, compared to the post-NAC early recurrent group as well as the paired pre-NAC nonrecurrent biopsies (Fig. 3). Tubulin beta 4A (*TUBB4A*) was the most frequent gene in these gene sets, present in at least eleven (18.0%) gene sets, whereas the pre-NAC expression levels for *TUBB4A* were not significantly different. Conversely, the second cluster contains 190 gene sets that were up regulated in the post-NAC nonrecurrent tumors. Furthermore, this cluster was downregulated in the post-NAC ERC cohort compared to the paired pre-NAC ERC sample biopsies. The heatmap image is presented with the labeled gene clusters, and the gene sets' most commonly represented genes are shown in the word clouds in Fig. 3. In summary, Cluster 1 gene sets downregulated in the post-NAC NRC patients were predominately associated with tubulin. In Cluster2, the up-regulated gene sets included growth receptors such as *EGFR*, *TGFBR2*, and *GHR*, as well as tumor suppressor genes found in apoptotic gene sets. Among the 660 DE genes between ERC and NRC at post-NAC, 97 were also identified in the 251 gene sets. Although the overlap is relatively small, gene sets identified (*n* = 6 with *p*-value < 0.05) by the GESA analysis on the 660 DE genes showed consistent directionality with those in the GSVA analysis (Additional file 2: Table s8b). It is also noted GSVA analysis identified more global changes compared to the GSEA analysis based solely on the DE genes.

### I-SPY1 confirmation of the core gene set signature from the BEAUTY TNBC cohort

We investigated the I-SPY1 study to validate our pathway analysis findings [13]. Like previously described, due to the platform differences between the I-SPY1 (microarray) and BEAUTY (RNA-Seq) datasets, we calculated the gene set scores for 188/251 (74.9%) gene sets using the ISPY-1 microarray data and GSVA method. Of the 188 gene sets, 56 gene sets (29.8%) demonstrated high similarity between the I-SPY1 and BEAUTY post-NAC studies (Additional file 2: Table s9 of 56 gene sets). Among these 56 gene sets, 17 gene sets (Cluster 1, Fig. 3) were down-regulated, and 39 (Cluster 2, Fig. 3) were up-regulated in the post-NAC I-SPY1 early-recurrence tumors. Cluster 1 could not recapitulate the same set of genes as TUBB4A was not assayed on the microarray platform used for the I-SPY trial. We observed five consistent genes in Cluster 2, including *ERLIN2, FCER1A, TGFBR2, PER2*, and *EGFR*. These genes are commonly included in multiple gene sets, such as *MYC* targets, *TGFβ, FOXO*, and *PI3K.*

### Identification of 17-gene signature to predict recurrence of cancer in chemoresistant tumors

**Fig. 4:**
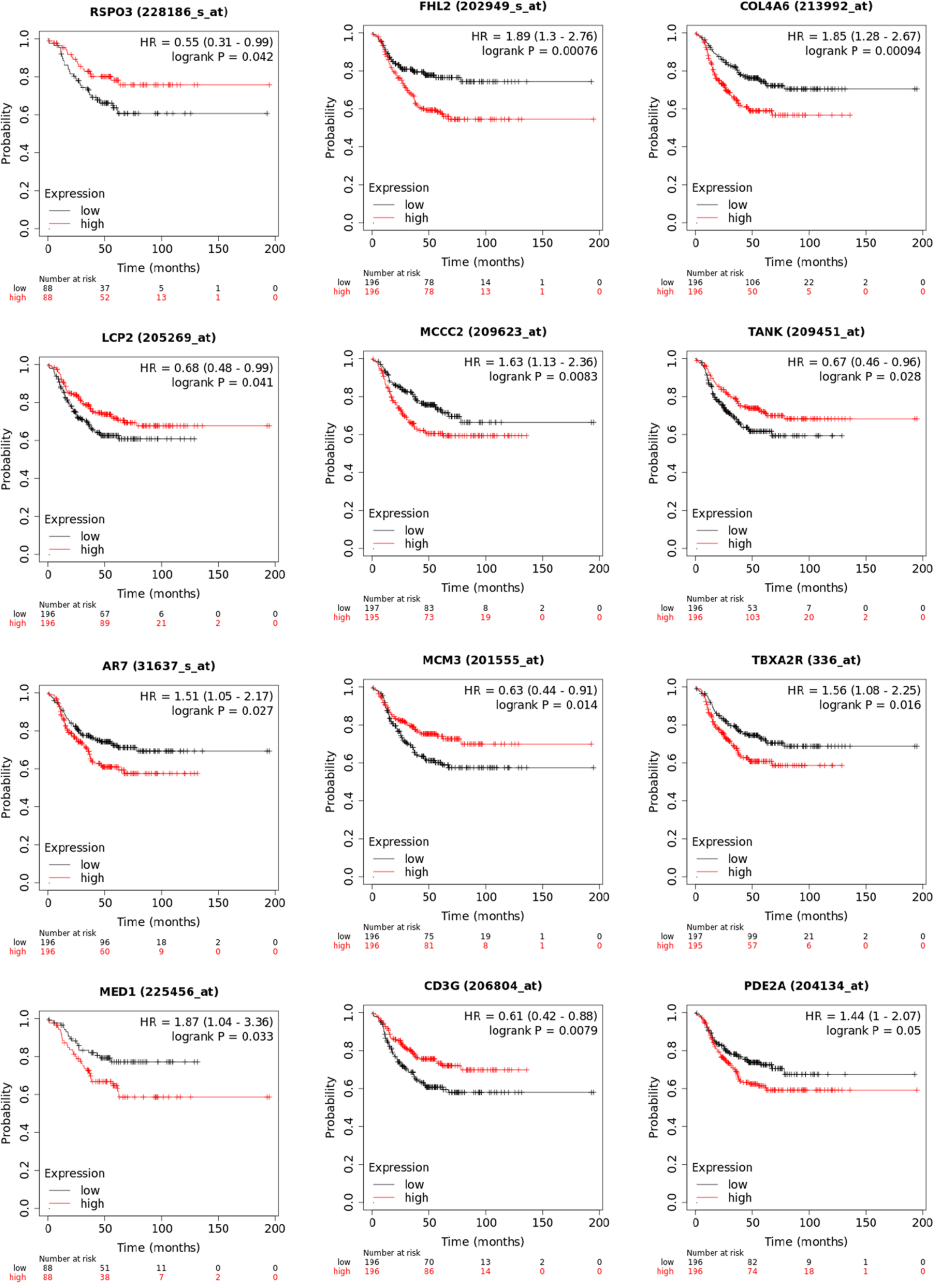
Seventeen genes were associated with survival analysis in an independent TNBC cohort (*n* = 392) from the KM plotter database. The first twelve of them, sorted according to their p values observed in the BEAUTY are presented in this plot. Additional plots can be found in the Additional file 1: Figure s2

We identified 113 differentially expressed genes that were up/down regulated in the same direction between the ERC and NRC patients in the I-SPY1 and BEAUTY studies related to 56 gene sets (listed in Additional file 2: Table s9). We assessed the predictive value of these 113 genes in a publicly available independent TNBC cohort (*n* = 392) using the Kaplan–Meier (KM) plotter online breast cancer database [38]. Our analysis shows that 17 genes were significantly associated (log-rank test *p*-value < 0.05) with relapse-free survival (RFS) in the TNBC cohort (Fig. 4, Additional file 1: Figure s2). Further investigation into this sub-cohort with positive lymph node disease (*n* = 189) showed that 10 of the 17 genes remained significant (Table 2). Additionally, we evaluated the proportional hazards for each gene in the BEAUTY and I-SPY1 trials. The hazard ratios between the two studies showed a significant correlation (Pearson correlation coefficient = 0.78) between the two studies (Additional file 2: Table s10).

**Table 2:** List of the 17 gene signature

Gene	Beauty logFC	Beauty *p*-value	ISPY logFC	ISPY *p*-value	logrank test *p*-value	logrank test (LN +) *p*-value	Location	Type(s)	Cytoband
RSPO3	− 2.32	0.00114	− 0.692	0.152	0.0422	0.05	Extracellular space	Kinase	6q22.33
FHL2	− 1.06	0.0014	− 0.345	0.518	0.0008	0.0004	Nucleus	Transcription regulator	2q12.2
COL4A6	− 3.27	0.00197	− 0.2	0.722	0.0009	0.019	Extracellular space	Other	Xq22.3
LCP2	1.82	0.00225	0.091	0.742	0.0407	0.22	Cytoplasm	Other	5q35.1
MCCC2	− 0.942	0.00356	− 0.118	0.73	0.0083	0.039	Cytoplasm	Enzyme	5q13.2
TANK	− 1.04	0.00396	− 0.275	0.622	0.028	0.17	Cytoplasm	Other	2q24.2
THRA (AR7)	− 1.32	0.00635	− 0.11	0.819	0.0266	0.19	Nucleus	Nuclear hormone receptor	17q21.1
MCM3	0.907	0.0111	0.478	0.343	0.0139	0.0017	Nucleus	Enzyme	6p12.2
TBXA2R	1.24	0.0119	0.088	0.812	0.0162	0.017	Plasma membrane	G-protein coupled receptor	19p13.3
MED1	− 0.676	0.0181	− 0.14	0.569	0.0333	0.077	Nucleus	Transcription regulator	17q12
CD3G	− 1.4	0.0211	− 0.506	0.133	0.0079	0.13	Plasma membrane	Transmembrane receptor	11q23.3
PDE2A	− 1.38	0.0313	− 0.301	0.435	0.0498	0.053	Cytoplasm	Enzyme	11q13.4
CITED2	− 1.11	0.032	− 1.403	0.046	0.0032	0.13	Nucleus	Transcription regulator	6q24.1
NR1D2	− 0.818	0.0358	− 0.591	0.236	0.022	0.014	Nucleus	Ligand-dependent nuclear receptor	3p24.2
AGGF1	− 0.606	0.039	− 0.626	0.099	0.0278	0.48	Cytoplasm	Other	5q13.3
RPS6KA4	0.744	0.0391	0.633	0.135	0.0147	0.017	Cytoplasm	Kinase	11q13.1
SNRPF	0.609	0.0492	0.743	0.072	0.0493	0.13	Nucleus	Other	12q23.1

Here we report the log fold change (logFC) and *p*-values observed in the BEAUTY and I-SPY1 cohorts. We also report the *p*-values obtained from the Kaplan–Meier plotter tool (https://kmplot.com) and annotations including genomic location, cellular location and functional type

### Prediction of recurrence using chemoresistant gene expression data, machine learning models, and cross-validation methods with the 17 gene signature

**Fig. 5:**
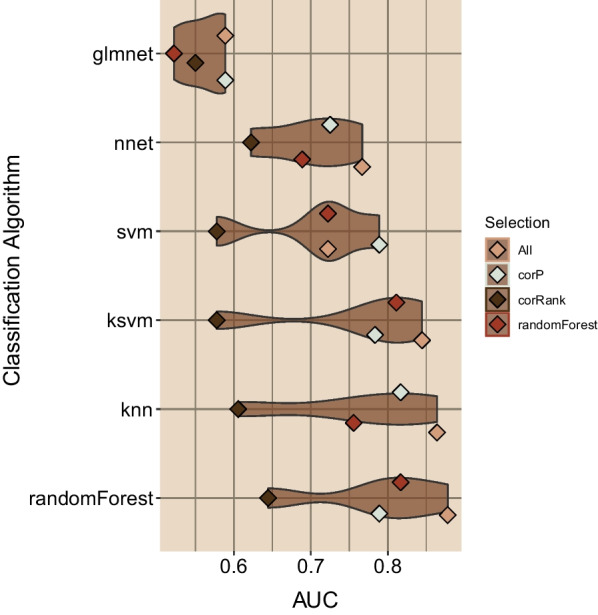
Classification assessment of seventeen genes associated with triple-negative breast cancer recurrence in chemoresistant tumors. Six classifiers were evaluated with the SuperLearner package employing seventeen genes and three downs-selection methods. The mean cross-validation AUC is plotted as a point for each classification method, and the four feature selection methods used are shown. We observed that three models, kernel support vector machine, kNN, and random forest models, achieved the best overall median classification AUC

Since there are no independent neoadjuvant breast cancer studies with publicly available data to validate our signature, transcriptomic data from the BEAUTY and I-SPY1 studies were pooled together to evaluate the 17-gene signature using machine learning models. We examined six classification algorithms, including a generalized linear model (glmnet), k nearest neighbors (k-NN), neural networks, random forests, support vector machine (SVM), and kernel SVM. The models were implemented as wrappers in the R package SuperLearner using gene down selection methods (Pearson's correlation, Spearman's rank correlation, and random forest). Given the lack of sampling power, we implemented a threefold cross-validation strategy, which we believe provided the most reasonable cross-validation strategy without subsampling the data into too trivial of a sample representation. We monitored the area under the receiver operating curve as an indicator of the model's classification performance, as shown in Fig. 5. The rank-based correlation (deep brown diamond) consistently underperformed other feature selection approaches (Additional file 1: Figure s3).

Most importantly, the ensemble of seventeen genes (labeled as 'All' in Fig. 5, tan diamond) was consistently the best-performing approach. We present the distribution of AUCs observed for each classification wrapper in Fig. 5. The models are ordered by increasing median AUC values, and kernel support vector machines, k nearest-neighbor (kNN), and random forest were the best performing modeling approaches, with the ensemble of all 17 genes achieving AUCs of 0.84, 0.88, and 0.88, respectively. A similar assessment of a model based on the available clinical features (as shown in Table 1) was also performed to compare with the transcriptome data-based 17-gene signature model. Due to the limited clinical data available for the I-SPY1 cohort, this comparison was limited to the BEAUTY TNBC cohort. The best performance of the clinical feature-based models was AUC = 0.69, and clinical feature selection failed to improve the performance of the clinical feature-based models. Overall, the 17-gene signature provided classification model that outperformed all clinical feature models.

*Differential expression of 17 genes in post-NAC TNBC ERC and NRC cohorts compared to paired pre-NAC tumors, primary tumor (baseline) samples from TCGA, and TCGA normal-adjacent tumors and the association with pCR.*

We investigated several datasets to confirm that the dysregulation of our signature's 17 genes was 1) specific to non-LAR tumors and 2) specific to post-NAC non-LAR tumor biopsies (Table 3). We evaluated the expression among eleven paired TNBC primary tumors (in LARs and non-LARs) and normal adjacent samples from TCGA and observed dysregulation among the tumor samples, with 9 out of the 17 (52.9%) surveyed being differentially expressed and 7 of 9 genes confirmed to be DE when the analysis was refined to the nine paired non-LAR TCGA tumors (Additional file 1: Figures s4-s5). Moreover, among these seven genes, we confirmed dysregulation in a core set of six genes (*COL4A6*, *AGGF1*, *TANK*, *CITED2*, *PDE2A*, and *MCM3*) in an independent cohort of *n* = 444 TNBC samples (Table 3, Additional file 1: Figure s6). These publicly available microarray datasets collated from the Thompson et al. 2022 study were primarily assayed with the Affymetrix U33 platform [17].

**Table 3:** Publicly available TNBC gene expression datasets that are included in this analysis for the 17-gene signature validation

TNBC	Study name	Total	LAR	Non-LAR	non-LAR			DE	Platform	References
					Non-pCR	pCR	Missing			
Tumor only	GSE106977	119	22	97	54	43	–	RD:pCR	Microarray	[39]
	GSE25065	60	11	49	27	18	4	RD:pCR	Microarray	[42]
	GSE25505	116	22	94	59	34	1	RD:pCR	Microarray	[55]
	GSE32646	26	6	20	12	8	–	RD:pCR	Microarray	[41]
Paired Tumor Normal	TCGA	11*	2*	9*	–	–	–	Tumor:Normal	RNA-Seq	[37]

^*^The number represents paired samples for the TCGA TNBC data

**Fig. 6:**
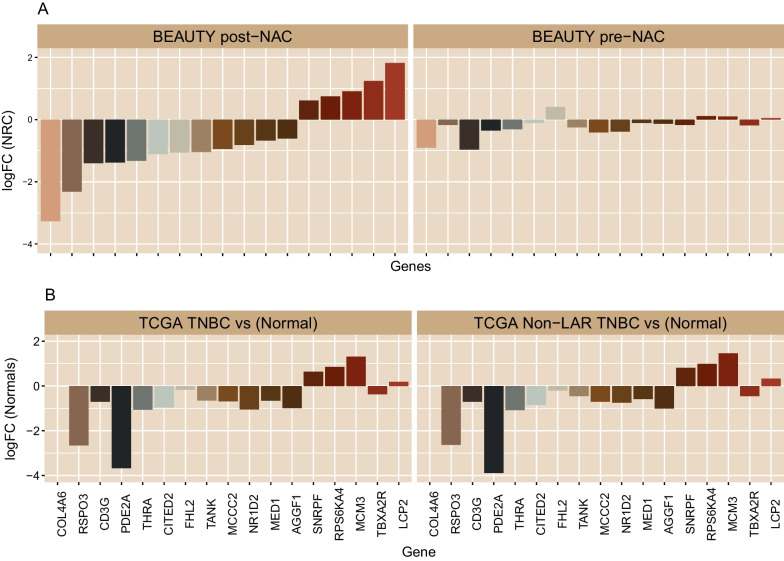
Expression profile of genes that distinguishes early recurrence in post-NAC biopsies. **A** BEAUTY expression profiles of the seventeen genes were observed to be differentially expressed with respect to the ERC in TNBC post-NAC biopsies (left panel). However, none of these genes were observed to be differentially expressed among the ERC in the pre-NAC biopsies (right panel). Four genes (FHL2, TANK, PDE2A, and RSPO3) are related to immune response signaling, and they were down regulated in the post-NAC samples of the ERC group. **B** TCGA TNBC expression profiles were confirmed to arise from tumor tissues compared to the paired normal-adjacent reference tissues. Bar plot shows the log fold changes (logFC) from all TCGA TNBC samples versus their paired normal adjacent tissues (left panel) Moreover, the expression profile was specific to TCGA non-LAR TNBC tumors as depicted by their logFC compared to respective paired normal adjacent samples (right panel). (Additional file 1: Figures s3-s6)

Furthermore, we evaluated whether dysregulated expression of these 17 genes existed prior to NAC treatment. As shown in Fig. 6, we verified that the differential expression of these 17 genes was specific to post-NAC tumors. In contrast, none of the genes were observed to be differentially expressed among the pre-NAC tumor biopsies. We subsequently investigated the 288 (of 388) non-LAR primary tumor TNBC samples from the independent cohort (124 pCR, 164 non-pCR, 100 missing). We observed that only 2 (12.5%) of the 16 genes (*RSPO3* was not assayed on the microarray platform) were differentially expressed (Additional file 1: Figure s7). In summary, the dysregulation of the 17 genes identified in this study was associated with post-NAC recurrence only in non-LAR TNBCs with residual disease after NAC (Additional file 1: Figure s8).

## Discussion

Triple-negative breast cancers are phenotypically heterogeneous diseases that lack therapeutic targets, and as such neoadjuvant chemotherapy is the standard of care. For 25–30% of patients who achieve pCR, the 3-year overall survival rate is 94%. However, for patients with residual disease after NAC, approximately 50% of patients will develop recurrent disease within the first 3–4 years [7]. We have previously reported the up regulation of genes (including *ERBB4*, *EGF*, *MAPK10*, *KIT*, and *FGFR2*) involved in hormone receptor HR cross-talk and the androgen receptor signaling pathway (*AR*, *FOXA1*, *FOXA2*, and *FOXA3*) associated with resistance to NAC treatment in TNBC patients using pre-NAC multiomics data [6]. Hence, to investigate potential biomarkers delineating early recurrence among TNBC NAC-resistant patients, we evaluated longitudinal TNBC tumor biopsies. To ensure a clean signal, we removed LAR samples and confirmed that post-NAC biopsies contained more than the tumor bed using an *in-silico* deconvolution model.

Our initial investigation of the ERC and NRC groups pre-NAC transcriptome gene expression identified only 11 genes to be differentially expressed ((FDR < 0.05). While analysis of pre-NAC RPPA data identified 20 differentially regulated proteins and 10 differentially regulated phosphorylation sites. Conversely, there was minimal change in the post-NAC RPPA data. The RPPA assay should be noted is constructed with a bias of signaling related proteins. This would suggest a transcriptional and translational temporal lag in signaling pathways, and that the post-NAC data reflects a common response to therapeutic intervention. However, the study of the transcriptional differences between ERC and NRC in the post-NAC transcriptome identified 660 genes which suggest that therapeutic selection pressure drives post-NAC differences. Among the 660 differentially expressed genes (FDR < 0.05 and |logFC|> 1), we observed the enrichment of genes in post-NAC data in a few cytoband regions (Fig. 1C-E, Fig. 2). Two of these regions (17q25 and 1q23-24) were confirmed to demonstrate copy gains in the pre-NAC biopsy, which coincided with the elevated expression observed in the post-NAC early recurrent samples (Fig. 2). Three genes in the 17q25 region are involved in sumoylation: *NUP85*, *CBX2*, and *CBX4*. Similarly, the cytoband 4p15 presented a copy loss in the early recurrent tumors (pre-NAC), and the post-NAC data also demonstrated decreased expression (not shown. Contrary to the transcriptional observations, the protein expression levels were more altered in the pre-NAC data (20 of 232 total proteins and 10 of 63 phosphorylation sites, *p*-value < 0.05). In contrast, four protein changes were observed in the post-NAC data.

A parallel track of analysis using topological gene set measurements was adopted to navigate the challenges we faced, given the relatively low sampling power. To reduce the number of genomic features (20,543 genes), we conducted a GSVA analysis (with 2282 gene sets) on the post-NAC BEAUTY gene expression data and leveraged our observations with the independent I-SPY1 study post-NAC microarray data. We observed that 251 gene sets were altered between the ERC and NRC BEAUTY TNBC tumors (Fig. 3). Cluster analysis of the 251 gene sets identified two clusters, with the first cluster including *TUBB4A*, the therapeutic target of paclitaxel. The gene sets which were up regulated in the ERC and included metastasis-promoting gene sets, DNA mismatch repair, and *TP53* gene sets. In contrast, the second cluster of gene sets that were down regulated in the ERC samples consisted of tumor suppressor gene sets, including *FOXO* signaling, *TGF-β* signaling, and apoptosis (Fig. 3).

Due to the technology differences between the microarray (I-SPY1) and RNA-Seq (BEAUTY) data, only 188/251 gene sets from the BEAUTY study were also investigated in the I-SPY1 study. We confirmed 56/118 gene sets in the I-SPY1 data to be significantly and concordantly altered, among which 113 genes were significantly and concordantly DE in both study cohorts. We refined the 113 genes by evaluating their individual prognostic value with the publicly available TNBC cohort provided by KM-plotter. We identified top candidate genes (*n* = 17) associated with recurrence-free survival in the KM-plotter TNBC cohort log-rank test *p*-value < 0.05 and adjusted *p*-value < 0.3 (Fig. 4, Additional file 1: Figure s2). Among these 17 genes, four genes (*FHL2*, *TANK*, *PDE2A*, and *RSPO3*) were well documented to be associated with breast cancer and down regulated in the post-NAC early recurrent samples. *PDE2A* is a phosphodiesterase that regulates mitochondrial respiration and mitogenic clearance [48, 49]. *FHL2* is a zinc finger transcription factor associated with several cancers, including ovarian and cervical cancers [50–52]. We note that the I-SPY1 trial observed an increase in interferon signaling associated with shorter recurrence-free survival among nonresponding patients [13]. Recent research has suggested that two molecules, *RSPO3*, and *TANK*, are related to *NF-κB* signaling and survival response through the induction of inflammatory molecules. *RSPO3* down regulation is involved in prostate cancer invasiveness and interacts with the inflammatory mediator *IL-1β *[53, 54]. Two SNPs (*rs17705608* and *rs7309)* in the *TANK* gene have been associated with breast cancer risk [55–57], involving TNF-mediated signal transduction. Most importantly, *TANK*, a member of the *TRAF* family, binds to *NEMO* (*IKKγ*) to induce inflammation through the *IKK* complex and *NF-κB* signal transduction [58]. These findings suggest that initiating inflammatory signaling via *NF-κB* signal transduction might be integral to recurrent free disease. Moreover, we observed significant down regulation (− 2.91, *p*-value 0.044) of the key apoptotic protein, *BCL2*, suggesting that inflammation might be accompanied by immune infiltration and subsequent cell death. We also compared this 17-gene signature of early recurrence with the DE genes for chemoresistant (pCR vs non-pCR) in the BEAUTY TNBC cohort previously described in [6]. We observed only one gene (*COL4A6*) presented in both DE analyses.

Cross-validation analysis of these 17 genes demonstrated a robust ability to predict recurrence, particularly with random forest, kNN, and kernel SVM algorithms (Fig. 5), achieving an AUC of 0.88. We investigated several datasets and confirmed that our 17 gene signature was specific to non-LAR tumors and post-NAC non-LAR tumor biopsies (Table 3). We also systematically analyzed the in-house and publicly available TNBC gene expression NAC datasets using computational biology and machine learning methods. We concluded that the post-NAC non-LAR TNBCs changed significantly during treatment compared to the baseline pre-NAC tumors. We have shown that the 17 genes identified in this study are novel biomarkers that predict recurrence in post-NAC residual tumors. Given the nature of our research, where we needed clinical, and omics data from paired pre- and post-NAC tumors from the same TNBC patient cohort, our ability to robustly evaluate the data was limited. However, we consciously tried to reduce the potential for false discoveries with our limited sampling population by applying *p*-value cutoffs along with fold change thresholds. Although we observed an average AUC of 0.88 using cross-validation analysis of the publicly available dataset and our Mayo Clinic BEAUTY study, further validation of the 17-gene signature in large TNBC cohorts is necessary.

## Conclusions

We observed notable differences in chemoresistant post-NAC TNBC tumor biopsies, significantly influencing recurrence-free survival. Among these transcriptional changes, we confirmed the dysregulation of a 17-genes signature specific to non-LAR TNBC tumors. These genes have been implicated in initiating inflammatory signaling via *NF-κB* signal transduction. While the gene signature and the specific role of these genes needs further validation, we have confirmed the detrimental impact of immune microenvironment depletion. Additional Post-NAC datasets are required to develop prognostic gene signatures with greater reliability. Moreover, we believe that further investigation of the inflammatory response to NAC treatment could facilitate the identification of clinical biomarkers and ultimately improve the therapeutic efficacy.

## Supplementary Information

**Additional file 1.** Supplementary figures.

**Additional file 2.** Supplementary Tables.

## Data Availability

All data used in this analysis are publicly available. Pre-NAC BEAUTY study data are available through dbGap accession ID phs001050.v1.p1. I-SPY1 data are available through the GEO database (GSE32603).
