# Supplementary material for: Integration of multiomics data shows down regulation of mismatch repair and tubulin pathways in triple-negative chemotherapy-resistant breast tumors
Source: Breast Cancer Res. 2023 May 24;25:57. doi: 10.1186/s13058-023-01656-x (PMC10207800; doi:10.1186/s13058-023-01656-x)

Integration of multi-omics data shows downregulation of mismatch repair, purin, and tublin pathways in AR-negative triple-negative chemotherapy-resistant breast tumors

Supplementary Materials

Supplementary Figure 1

Three Venn diagrams demonstrating the limited concordance in gene level changes in expression between the I-SPY1 and BEAUTY clinical trials. A) Demonstrates post-NAC differential expression between early recurrent (ERC) and non Recurrent (NRC). B) The differential expression between pre-NAC and post-NAC among the ERC. C) The differential expression between pre-NAC and post-NAC among the NRC.

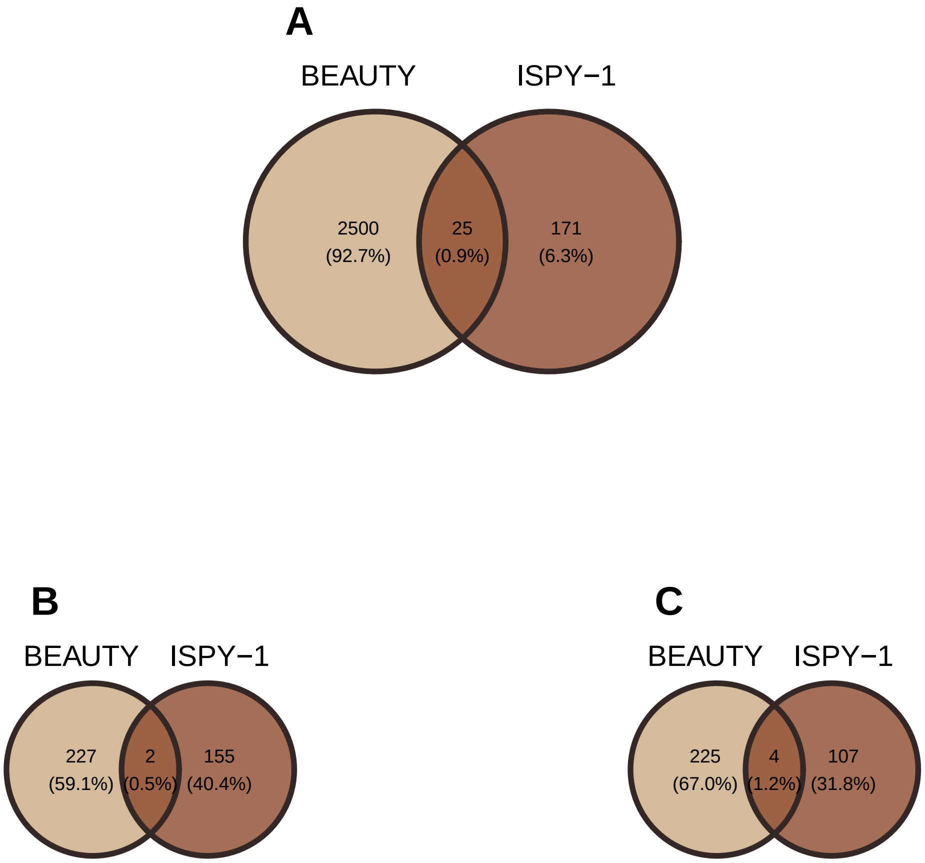

Supplementary Figure 2

Seventeen genes were observed to be associated with survival analysis in an independent TNBC cohort (N=392) from the KM plotter database. The first 12 are presented in the manuscript, ordered by significance. The remaining 5 curves are provided here and also ordered by pvalue.

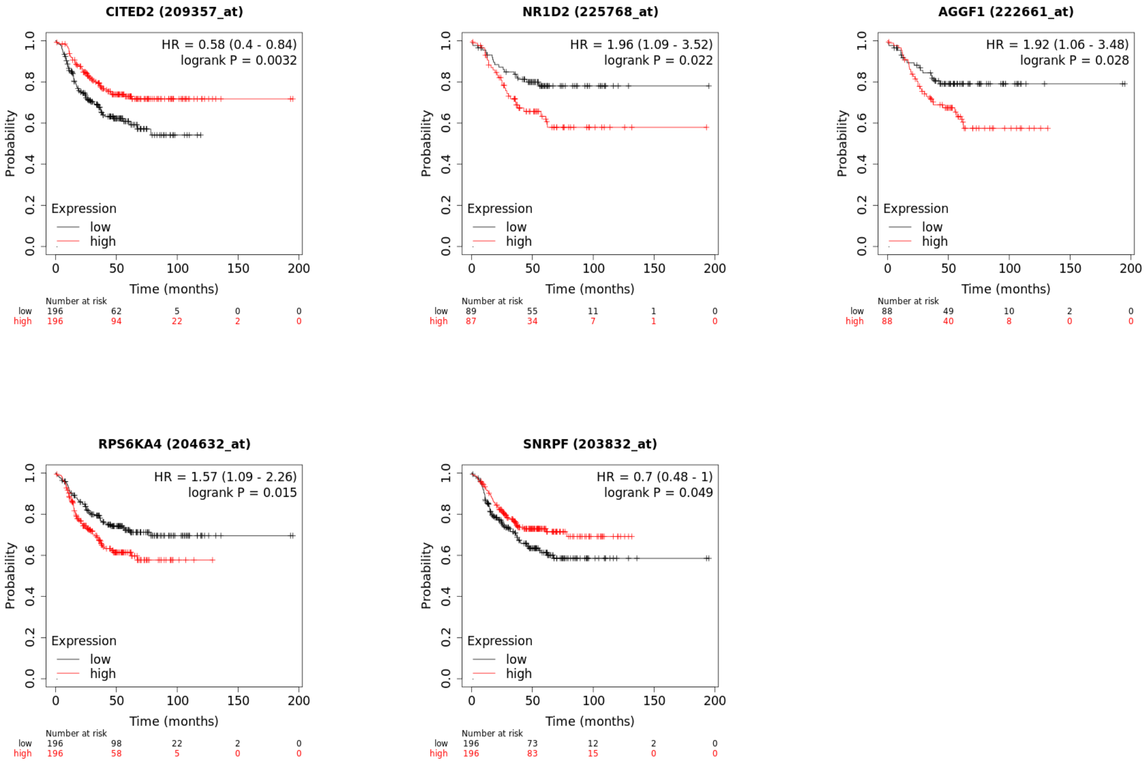

Supplementary Figure 3

Classification assessment of seventeen genes associated with triple-negative breast cancer recurrence in chemoresistant tumors. The mean cross-validation AUC is plotted as a point for each down selection method (including All or no down selection), and the six classification models are shown. We observe that Spearman's rank-based correlation consistently under-performed, regardless of the classification model. We also observed that general linear models (GLM) consistently under perform in classifications.

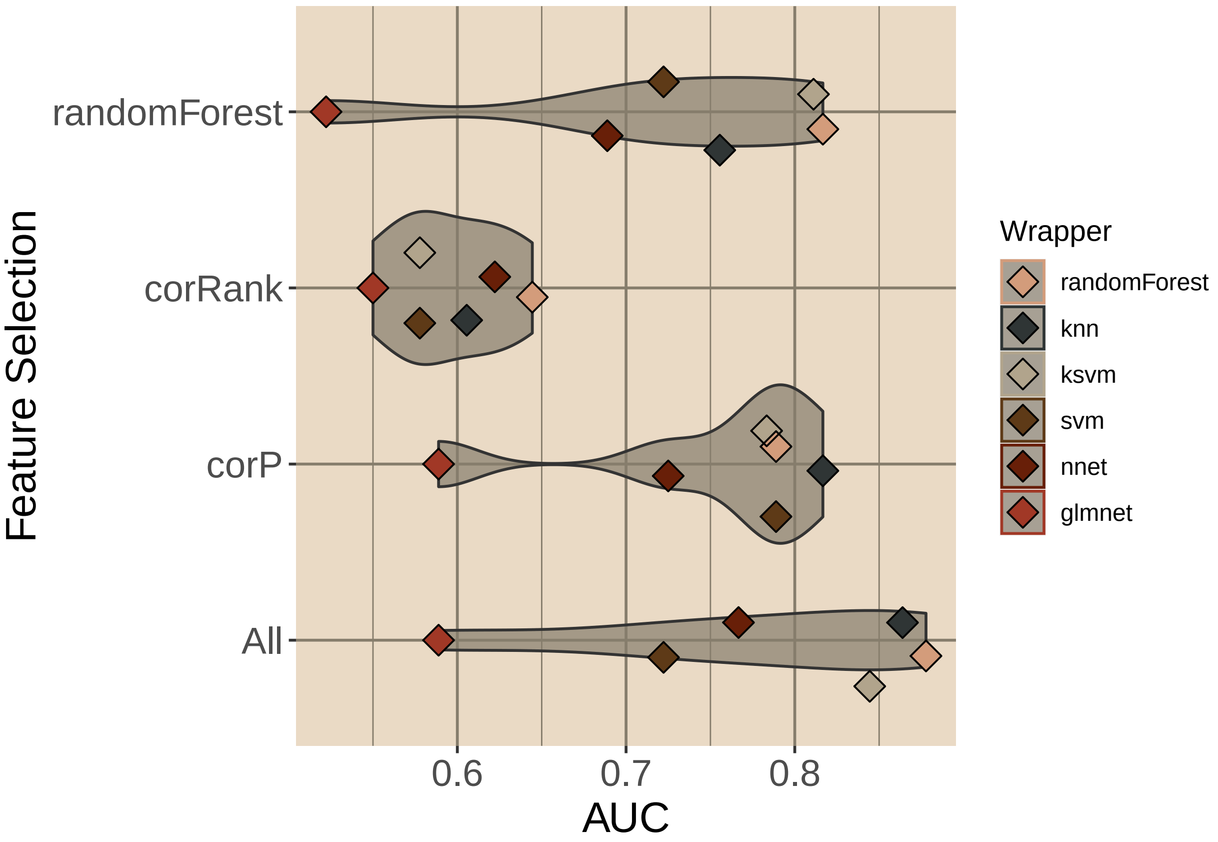

Supplementary Figure 4

Expression profiles of the 17 genes in the 11 normal-adjacent paired TCGA TNBC samples. Differential Expression was observed in 9 of the 17 (52.3%). The change (logFC) is presented in the right most bar graph. Brown indicates higher expression in the Normal (reference) and blue indicates higher expression in the TNBC (case).

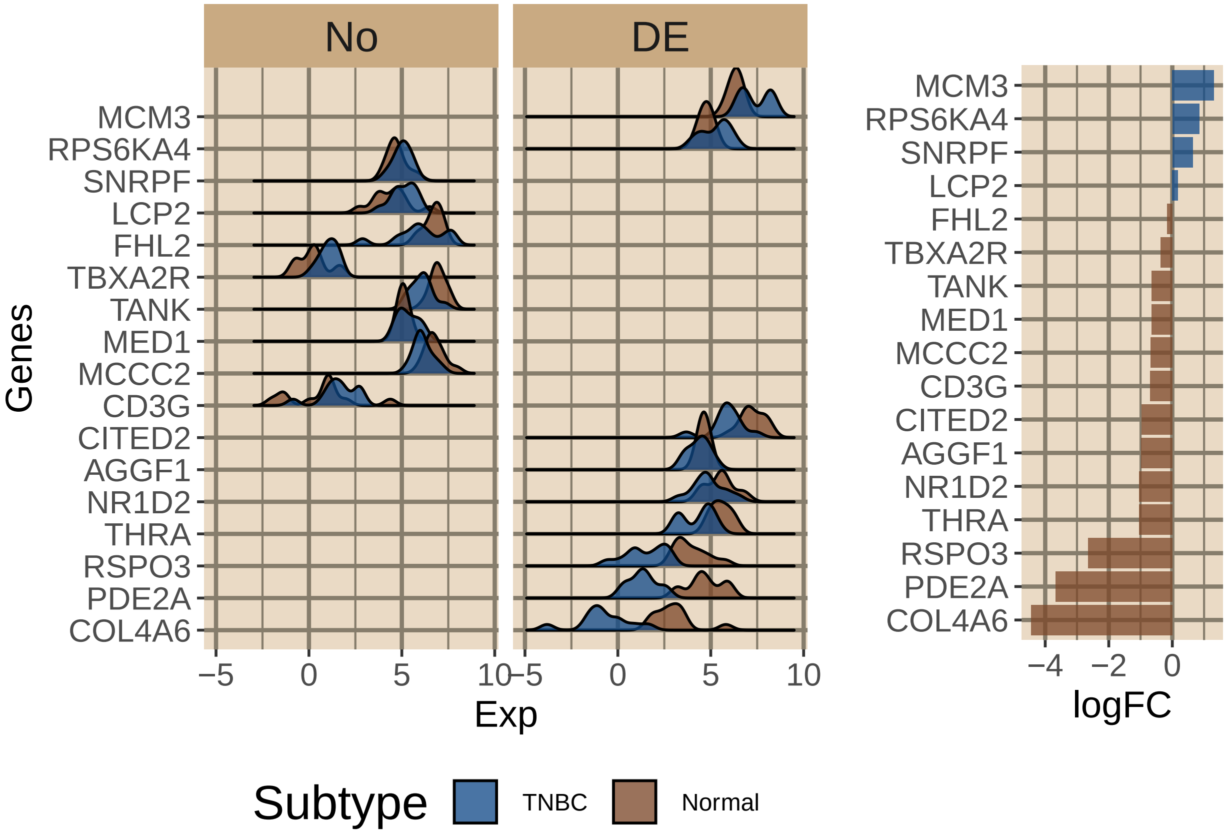

Supplementary Figure 5

Expression profile of the 17 genes in the 9 normal-adjacent paired TCGA Non-LAR TNBC samples. Two LAR samples were removed, along with their paired adjacent tissue sample. Differential Expression was observed in 9 of the 17 (52.3%). Eight of the genes observed as differentially expressed were also observed as differentially expressed in Supplementary Figure 3. The change (logFC) is presented in the right most bar graph. Brown indicates higher expression in the Normal (reference) and blue indicates higher expression in the Non-LAR (case).

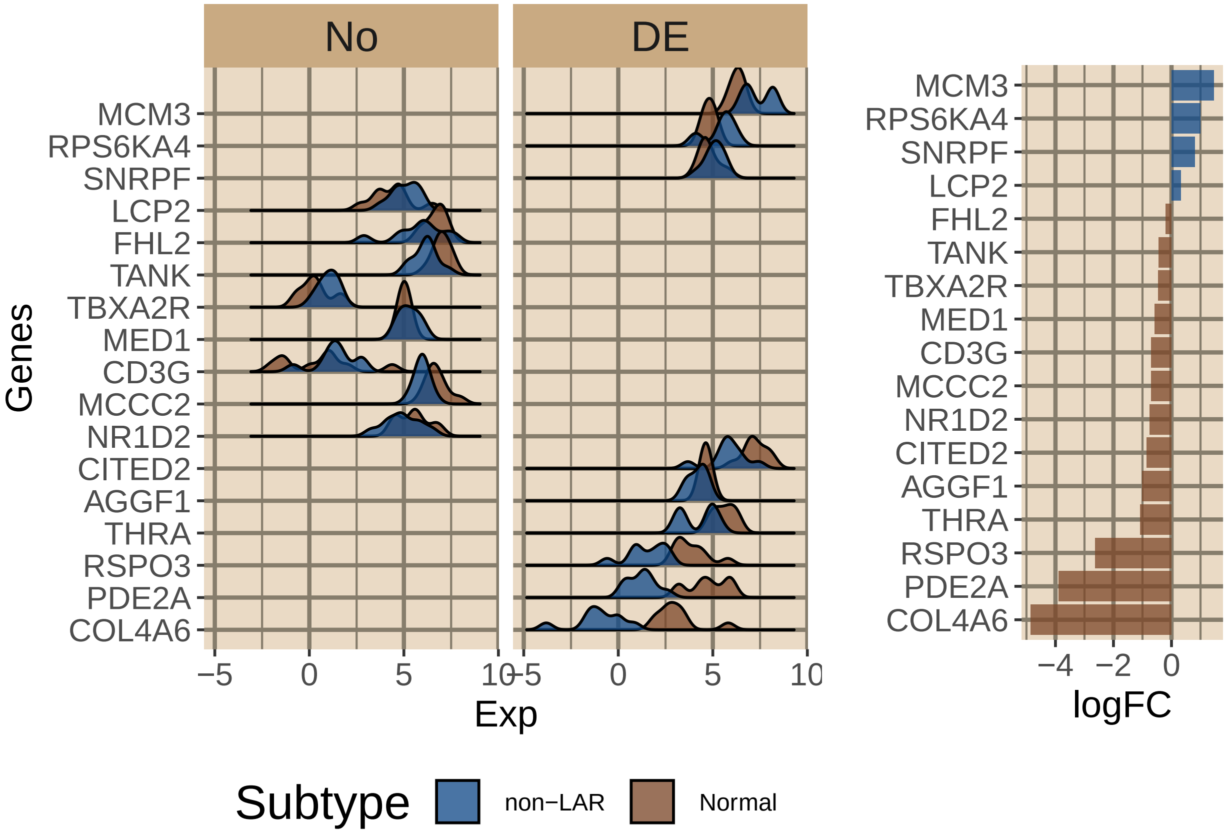

Supplementary Figure 6

Expression profile of the 16(17) genes of a scaled and combined TNBC cohort, consisting of 486 samples. There are 98 LAR and 388 Non-LAR TNBC samples. Differential Expression was observed in 9 of the 16 (56.3%) genes. The change (logFC) is presented in the right most bar graph. Brown indicates higher expression in the LAR (reference) and blue indicates higher expression in the Non-LAR (case).

RSPO3 was not observed in the combined dataset.

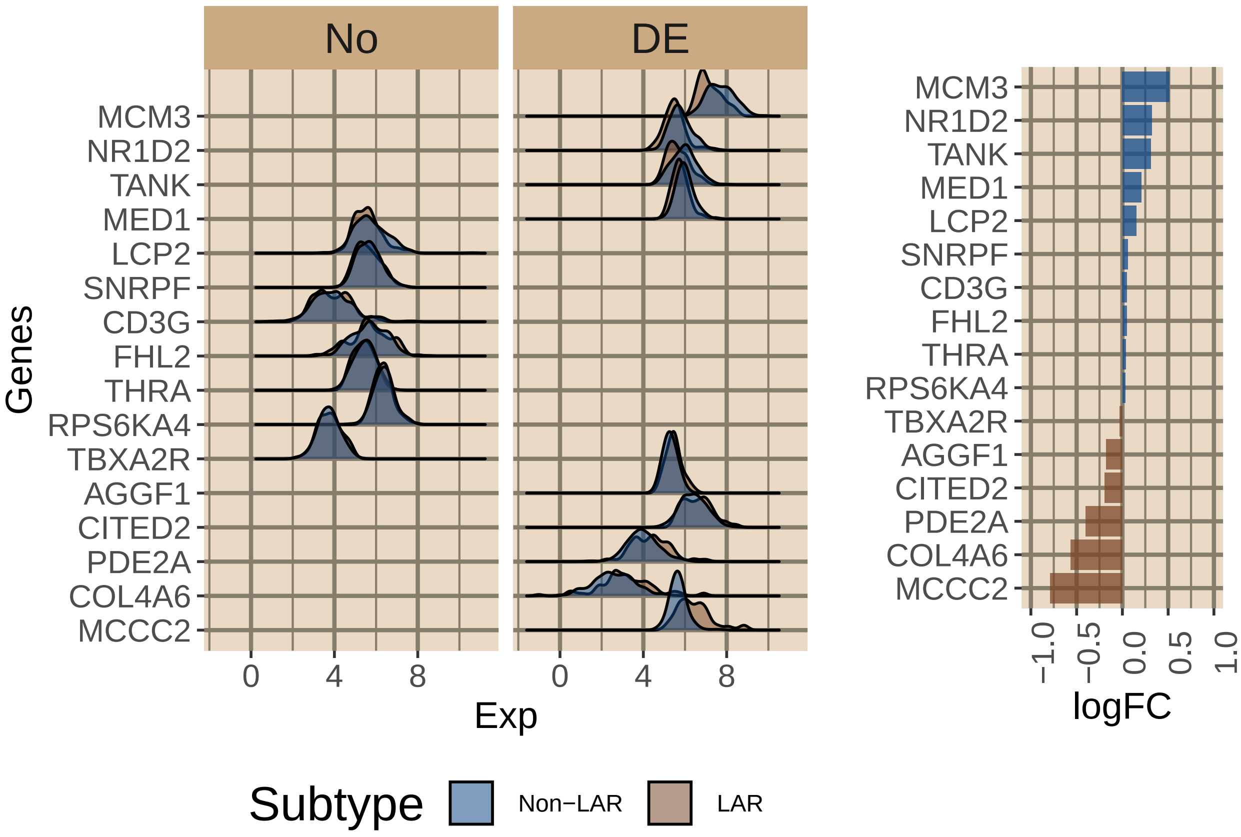

Supplementary Figure 7

Expression profile of the 288 Non-LAR TNBC samples from the scaled and combined, all of which received neoadjuvant treatment. Differential expression was evaluated between the 164 who failed to respond to the therapy and 124 patients which demonstrated pathological complete response. The change (logFC) is presented in the right most bar graph. Brown indicates higher expression in the pCR and blue indicates higher expression in the RD.

Only 2 of the 16 genes (12.5%) were observed to be differential expressed.

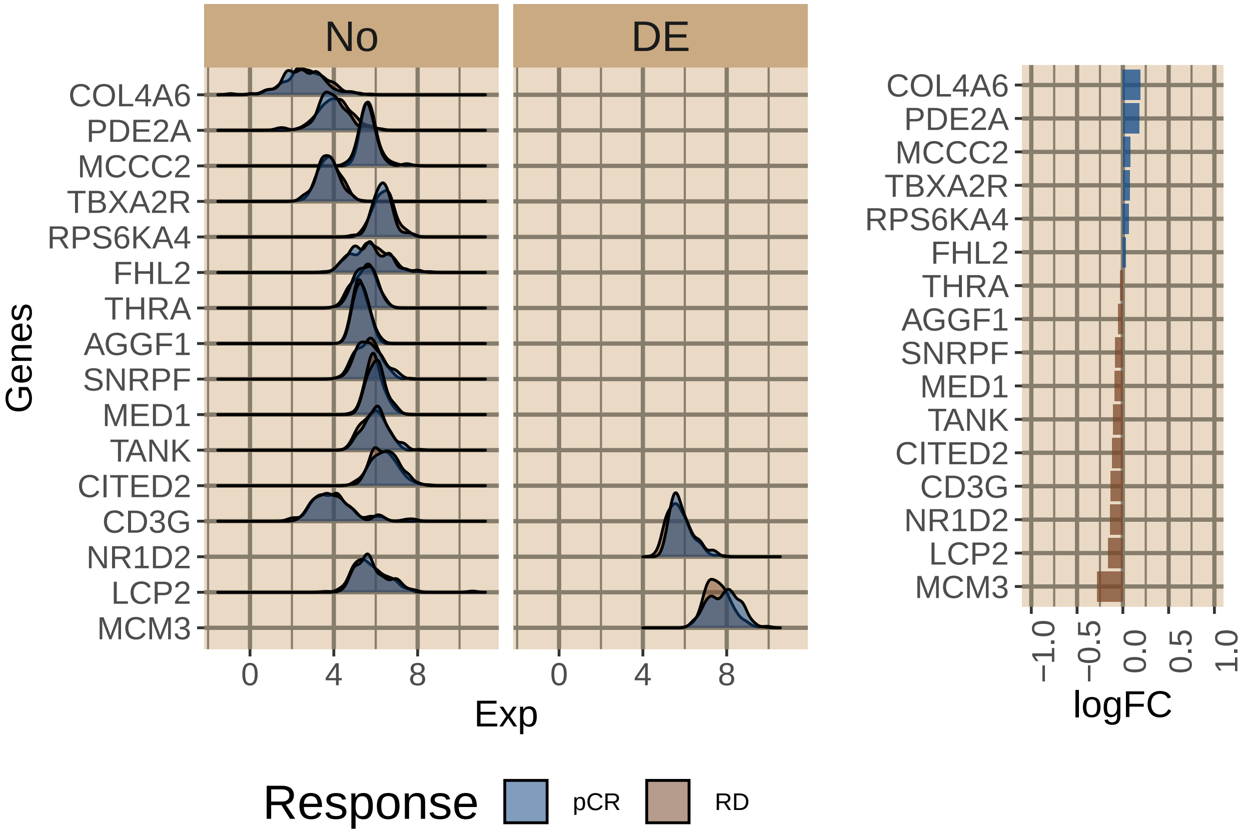

Supplementary Figure 8

Summary of the differential expression observed in the independent TNBC data. The left panel presents the observed log fold changes. We observe that the expression profiles is mostly reproduced in comparison to normal adjacent tissue (top row) and is arising from Non-LAR TNBC tissues. The bottom row presents the log fold changes from the scaled data. The profile is partially replicated in comparison to LAR TNBC tissues, but was not associated with pathological complete response. The right panel present the significance of the obseved changes in expression, following the same ordering. Although, The observed log fold changes were considerably smaller in the scaled dataset, we observed that the changes particularly between the LAR and Non-LAR TNBC were the most significant.

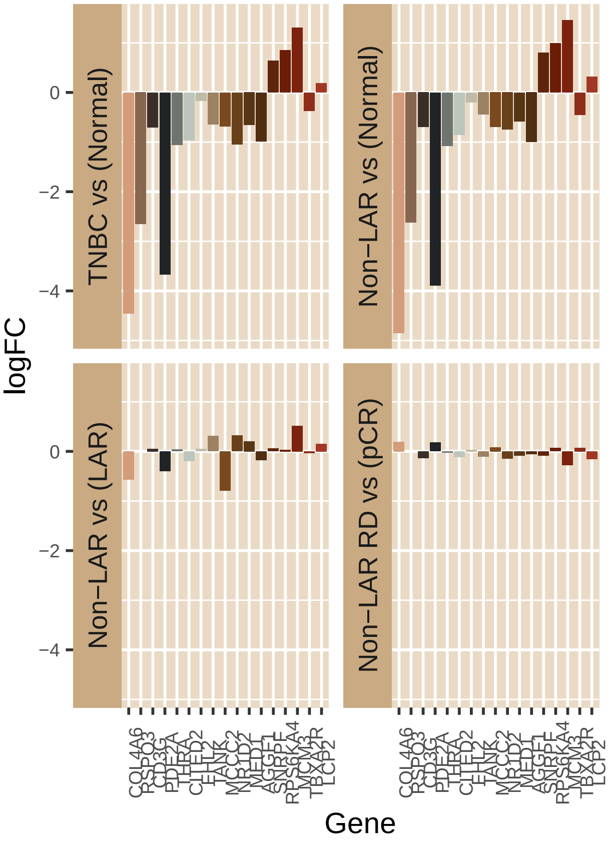

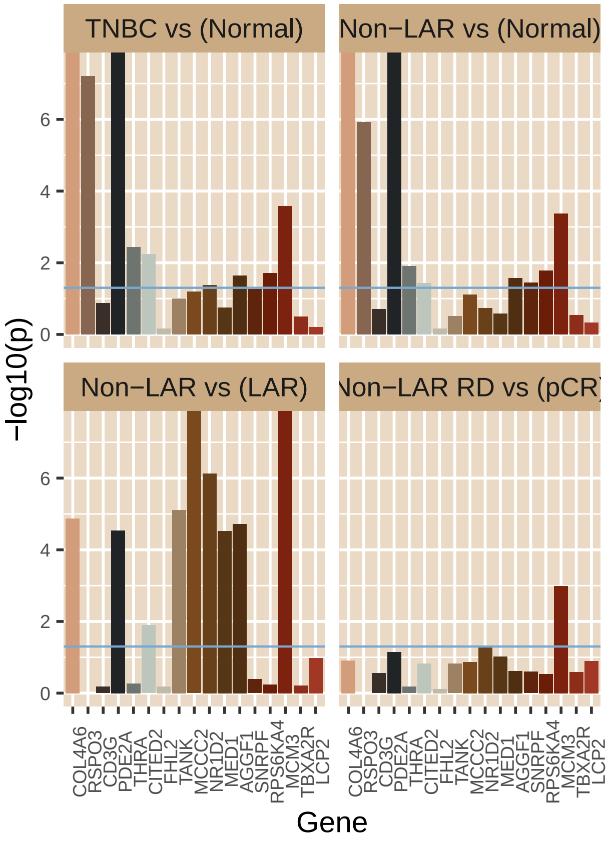

Supplement: Supplementary file 1 — Additional file 1. Supplementary figures. [file 13058_2023_1656_MOESM1_ESM.pdf]
